# The Aftermath of War

**Published:** 1920-07-31

**Authors:** 


### The Aftermath of War.

In his address to the Royal Sanitary Institute Sir Frederick Mott laid down the dictum that no real progress can be made in the study of the mind, in health and disease, if either the mental or the bodily part of man's nature is studied apart, one to the exclusion of the other; for they are indissolubly united. In the aftermath of the war he has been astonished at the ignorance of many of his professional brethren as to the functional disorders of those suffering from the effects of shell-shock, hysteria, and other war neuroses.

"At the Maudsley Hospital," he said, "we have cured hundreds of soldiers and pensioners in a few minutes, a few hours, or a few days, who had been in hospitals and convalescent homes months or years, and who, owing to ignorance of diagnosis, had been treated for organic disease by massage and electricity, carried out frequently by sympathetic ladies on the 'poor dear' principle, under the direction of medical practitioners unacquainted with neurological and psychological medicine." The war has produced no new nervous disease; it is the same hysteria and neurasthenia which neurologists knew before the war, and which were curable by the same methods. A fact brought home to all those who have had the care of war psycho-neuroses is that the signs and symptoms of hysteria and neurasthenia of men who have been to the Front and have been invalided home (except those in which there has been definite evidence of cerebral or spinal commotion or discussion, burial or gassing) in no way differ essentially from the signs and symptoms of hysteria and neurasthenia of soldiers who have never been out of England. In support of this statement Sir Frederick Mott mentioned that he had seen both hysteria and neurasthenia arise from the fear of conscription, or, having been conscripted, an hysterical crisis, contracture or paralysis has occurred when it became known that the conscript would be in a draft for general service abroad.

### The Doctor's Responsibility.

Even inoculation has been followed by hysterical paralysis, eventually cured by contra-suggestion. At the same time it is admitted that emotional as well as commotional shock due to terrifying or horrifying conditions of the war, may have induced hysterical manifestations in a neuropotentially sound individual. The anxiety neuroses and psychasthenia with obsessions which are still affecting so many people, both in military and civil life, are neuropathic functional disorders, which cannot be rapidly cured by suggestion and persuasion. The sufferers with these functional disorders are frequently quick-brained, intellectual men and women in all grades of society, and these, says Sir Frederick, are suitable subjects for psychotherapy. The doctor who has a knowledge of psychological medicine and makes it his business to study the desires and motives which determine conduct, and who is able to study that sympathetic relation with his patients whereby he gains their confidence, is able to uproot from the mind an anxiety causing a mental conflict, for which the patient is continually but unavailingly striving to make adjustments. The continuous mental conflict from which the patient affected by an anxiety neurosis suffers, goes on during sleep, exhausting his nervous energy and leaving him unrefreshed, depressed, and despondent on awakening.

This mental conflict which the experiences of the war and the dreams of soldiers of their terrible experiences has brought home to us in a vivid manner, reacts unfavourably upon all the bodily functions; then a vicious circle is established in which the *joie de vivre* can only be restored by removing the cause of the anxiety and improving bodily health. Now that the terrors of the war are passing away, we have the persistence of a widespread anxiety, neurosis occasioned by a contemplative fear for the future of self and family, in a large number of officers and men with an inadequate or deferred pension and no employment.

